# A MicroRNA-Based Network Provides Potential Predictive Signatures and Reveals the Crucial Role of PI3K/AKT Signaling for Hepatic Lineage Maturation

**DOI:** 10.3389/fcell.2021.670059

**Published:** 2021-06-01

**Authors:** Xicheng Wang, Wencheng Zhang, Yong Yang, Jiansong Wang, Hua Qiu, Lijun Liao, Tsunekazu Oikawa, Eliane Wauthier, Praveen Sethupathy, Lola M. Reid, Zhongmin Liu, Zhiying He

**Affiliations:** ^1^Institute for Regenerative Medicine, Shanghai East Hospital, School of Life Sciences and Technology, Tongji University School of Medicine, Shanghai, China; ^2^Shanghai Engineering Research Center of Stem Cells Translational Medicine, Shanghai, China; ^3^Shanghai Institute of Stem Cell Research and Clinical Translation, Shanghai, China; ^4^The First Affiliated Hospital of Nanchang University, Nanchang, China; ^5^Department of Traumatology, Shanghai East Hospital, Tongji University School of Medicine, Shanghai, China; ^6^Department of Anesthesiology and Pain Management, Shanghai East Hospital, Tongji University School of Medicine, Shanghai, China; ^7^Division of Gastroenterology and Hepatology, Department of Internal Medicine, Jikei University School of Medicine, Tokyo, Japan; ^8^Department of Cell Biology and Physiology, UNC School of Medicine, Chapel Hill, NC, United States; ^9^Department of Biomedical Sciences, Cornell University College of Veterinary Medicine, Ithaca, NY, United States

**Keywords:** microRNAs, PI3K/AKT signaling, biliary tree stem cells, hepatic lineage, let-7 family

## Abstract

**Background:**

Functions of miRNAs involved in tumorigenesis are well reported, yet, their roles in normal cell lineage commitment remain ambiguous. Here, we investigated a specific “transcription factor (TF)-miRNA-Target” regulatory network during the lineage maturation of biliary tree stem cells (BTSCs) into adult hepatocytes (hAHeps).

**Method:**

Bioinformatic analysis was conducted based on our RNA-seq and microRNA-seq datasets with four human hepatic-lineage cell lines, including hBTSCs, hepatic stem cells (hHpSCs), hepatoblasts (hHBs), and hAHeps. Short time-series expression miner (STEM) analysis was performed to reveal the time-dependent dynamically changed miRNAs and mRNAs. GO and KEGG analyses were applied to reveal the potential function of key miRNAs and mRNAs. Then, the miRDB, miRTarBase, TargetScan, miRWalk, and DIANA-microT-CDS databases were adopted to predict the potential targets of miRNAs while the TransmiR v2.0 database was used to obtain the experimentally supported TFs that regulate miRNAs. The TCGA, Kaplan–Meier Plotter, and human protein atlas (HPA) databases and more than 10 sequencing data, including bulk RNA-seq, microRNA-seq, and scRNA-seq data related to hepatic development or lineage reprogramming, were obtained from both our or other published studies for validation.

**Results:**

STEM analysis showed that during the maturation from hBTSCs to hAHeps, 52 miRNAs were downwardly expressed and 928 mRNA were upwardly expressed. Enrichment analyses revealed that those 52 miRNAs acted as pluripotency regulators for stem cells and participated in various novel signaling pathways, including PI3K/AKT, MAPK, and etc., while 928 mRNAs played important roles in liver-functional metabolism. With an extensive sorting of those key miRNAs and mRNAs based on the target prediction results, 23 genes were obtained which not only functioned as the targets of 17 miRNAs but were considered critical for the hepatic lineage commitment. A “TF-miRNA-Target” regulatory network for hepatic lineage commitment was therefore established and had been well validated by various datasets. The network revealed that the PI3K/AKT pathway was gradually suppressed during the hepatic commitment.

**Conclusion:**

A total of 17 miRNAs act as suppressors during hepatic maturation mainly by regulating 23 targets and modulating the PI3K/AKT signaling pathway. The regulatory network uncovers possible signatures and guidelines enabling us to identify or obtain the functional hepatocytes for future study.

## Introduction

Human biliary tree stem cells, hBTSCs, are the stem cells located in the peribiliary glands in the biliary tract (Miyajima et al., 2014). The isolation of hBTSCs and their *in vitro* and *in vivo* characterization indicate that they are stem cells of mature hepatocytes, cholangiocytes, and pancreatic endocrine cells. hBTSCs are found throughout the major duct of the biliary tree in all ages of donors, with the potential of giving functional cell types during the organ injury (Kaneko et al., 2015). At the distal portion of the bile duct in the liver parenchyma, the so-called Canals of Hering are known as niches where human hepatic stem cells, hHpSCs, are located (Gupta, 2000; Deng et al., 2018). Both hBTSCs and hHpSCs are successfully applied to rescue animals with liver dysfunctional diseases, and patients with end-stage liver diseases. However, as what has been discovered in the studies of generating hepatocytes from pluripotent stem cells, including embryonic stem cells (ESCs) and induced pluripotent stem cells (iPSCs), even under the precise stepwise differentiation strategies, cellular heterogeneity exists in stem cells derived hepatocytes, which often results in less functional hepatocytes compared to primary hepatocytes. Therefore, the urgency of understanding the mechanism underlying the hepatic maturation of hBTSCs and/or hHpSCs has been a fundamental task which enables the successful conduction of stem-cells-based liver disease therapies.

MicroRNAs (miRNAs), about 22 nucleotides long, regulate messenger RNA (mRNA) by either cleaving mRNA molecules or inhibiting their translation via binding to complementary regions in their 3′ untranslated regions (UTRs) to form RNA-induced silencing complexes. With these antisense mechanisms, miRNAs participate in diverse developmental and biological cellular processes, including stem cell differentiation and cell cycle regulation (Chen and Lodish, 2005; Chivukula and Mendell, 2008). Meanwhile, miRNAs exert their regulatory functions by targeting various genes pertaining to one or more signaling pathways, including the HIPPO, WNT/β-catenin, and PI3K/AKT signaling pathways. Taken what have been revealed in Li et al.’s (2020) study recently as example, miR-490-5p, an effective inhibitor of the metastasis of hepatocellular carcinoma, plays a negative role in the chondrogenic differentiation of human adipose-derived stem cells. This inhibition of miR-490-5p during chondrogenesis could promote the maintenance of cartilage phenotype mainly via the activation of PI3K/AKT signaling (Li et al., 2020).

Key regulatory functions of miRNAs in hepatocytes and in the liver formation during the embryonic development have also been partly studied (Hand et al., 2009; Laudadio et al., 2012). Knocking-down Dicer1, the enzyme essential for the processing of microRNAs, leads to miRNA depletion in the liver and, thereafter, results in an over-expression of fetal stage-specific genes and a promotion of hepatocyte proliferation (Sekine et al., 2009). On the other hand, the overexpression of miRNA-199a-5p negatively modulates the liver repopulation ability of ESC-derived hepatic cells strengthening the universal suppressing mechanism that miRNAs play (Mobus et al., 2015). Knocking-down of miR-23b in a fetal liver stem cell line not only inhibits their hepatocytic differentiation, but also promotes the expression of bile duct related genes, indicating a much more complicated regulatory function miRNAs play in liver cell fate determination (Rogler et al., 2009). However, the detailed regulatory network and how miRNAs manipulate the lineage maturation from stem cells at early lineage stages, including hBTSCs, toward hepatocytes remain to be unveiled.

In this study, we attempt to clarify the specific miRNA and mRNA regulatory network for determining the hepatic lineage maturation of hBTSCs into hAHeps. Although the hepatic differentiation of hBTSCs happens robotically when the requirements mechanism of functional hepatocytes is activated during the liver repair post-injury, the exact triggers and regulators to promote and persist the differentiation of hBTSCs remain uncovered. With the microRNA-seq and bulk RNA-seq data sequenced of four stages of hepatic lineage, including hBTSCs, hHpSCs, hHBs, and adult hepatocytes (hAHeps), it is possible for us to reveal the potential “TF-miRNA-target” orchestrated network that regulates the maturation of hepatocytes.

## Materials and Methods

### Human Biliary Tree

Human fetal livers and biliary tree tissue were obtained by elective terminations of pregnancy and provided by an accredited agency, Advanced Biosciences Resources (ABR). Tissues used in the experiments were from fetuses between 17 and 19 weeks. The research protocol was reviewed and approved by the Institutional Review Board (IRB) for Human Research Studies at the University of North Carolina at Chapel Hill.

Human fetal liver extrahepatic biliary tree tissues (gall bladder, common duct, hepatic ducts) were detached from the liver parenchyma. These were washed with the “cell wash” buffer comprised of a sterile, serum-free basal medium supplemented with antibiotics, 0.1% serum albumin, and 1 nM of selenium (10^–9^ M). Biliary tree tissue and liver parenchyma were processed separately following the same protocol. After the mechanical dissociation with crossed scalpels, tissue aggregates were enzymatically dispersed into a cell suspension in RPMI-1640 supplemented with 0.1% bovine serum albumin (BSA), 1 nM of selenium, 300 U/ml of type IV collagenase, 0.3 mg/ml of deoxyribonuclease (DNAse), and antibiotics. Digestion was done at 32°C with frequent agitation for 30–60 min. Most tissues required two rounds of digestions followed by centrifugation at 1,100 rpm at 4°C. Cell pellets were combined and re-suspended in the cell wash. The cell suspension was centrifuged at 30 G for 5 min at 4°C to remove the red blood cells. The cell pellets were again re-suspended in the cell wash and filtered through a 40 μm nylon cell strainer (Becton Dickenson Falcon #352340) and with a fresh cell wash. The cell numbers were determined, and viability was assessed using Trypan Blue. Cell viability above 90–95% was routinely observed. Colonies used for RNA-seq with the typical hBTSCs or hHpSCs morphologies formed within 3 weeks under the serum free Kubota’s medium culture. hHBs could be distinguished by the expression of ICAM-1 versus NCAM.

### MicroRNA-Sequencing and RNA-Sequencing Analysis

Total microRNA was isolated according to the Total RNA Purification Kit (Norgen Biotek, Thorold, ON, Canada) following the manufacturer’s instructions. microRNA integrity was quantified with the Agilent 2100 Bioanalyzer or 4200 Tapestation (Santa Clara, CA, United States). Libraries were generated utilizing the CleanTag Small RNA Library Prep kit produced by TriLink Biotechnologies (San Diego, CA, United States). In addition, sequencing was conducted on the platform of Illumina HiSeq2000 (San Diego, CA, United States).

Messenger RNA was purified using the Qiagen RNeasy Kit from the adult liver and biliary tree tissue and from isolated cell suspensions of hBTSCs, hHpSCs, hHBs, and hAHeps. RNA integrity analysis was performed using an Agilent 2000 Bioanalyzer. The cDNA libraries were prepared using the Illumina TruSeq Stranded mRNA preparation kit and sequenced on the Illumina HiSeq 2500 platform. More detailed information has also been shown in our previous published work (Oikawa et al., 2015; Dinh et al., 2019).

### Data Source

All 15 datasets, including our and other bulk RNA-seq, scRNA-seq, and microRNA-seq data, were collected from the Gene Expression Omnibus (GEO) database with processed series matrix files^1^. Six of our previous published datasets were used, including GSE73114 (Oikawa et al., 2015), GSE114974 (Dinh et al., 2019; Dinh et al., 2020), GSE101133 (Yan et al., 2017), GSE75141 (Wu et al., 2017), GSE105019 (Fu et al., 2019), and GSE116113 (Fu et al., 2019). Nine datasets from other teams were all used to validate the results in this work. The datasets are as follows: GSE57833, GSE57878, GSE90047 (Yang et al., 2017), GSE132034 (Gong et al., 2020), GSE28892 (Shin et al., 2011), GSE56734 (Ito et al., 2014), GSE25048 (Kim et al., 2011), GSE112330 (Xie et al., 2019), and GSE124528 (Wang et al., 2019). All the data have been normalized and exhibited in a heatmap by using the pheatmap R package^2^. The rows of heatmap were all scaled to better visualize the difference and expression changing pattern. The detailed information about these datasets are in Table 1.

**TABLE 1:** Detailed information of 15 datasets analyzed in this work.

Dataset	Year	Species	Platform	Data type	Team
GSE73114	2015	*Homo sapiens*	GPL16791 Illumina HiSeq 2500	Bulk RNA-seq	Oikawa et al., 2015
GSE114974	2019	*Homo sapiens*	GPL11154 Illumina HiSeq 2000	MicroRNA-seq	Dinh et al., 2019
GSE57833	2014	*Homo sapiens*	GPL18712 miRCURY LNA microRNA Array	MicroRNA-seq	Gallagher et al., 2014
GSE57878	2014	*Homo sapiens*	GPL14877	mRNA-seq	Gallagher et al., 2014
GSE90047	2017	*Mus musculus*	GPL13112 GPL17021	Bulk RNA-seq	Yang et al., 2017
				ScRNA-seq	
GSE132034	2020	*Mus musculus*	GPL13112 Illumina HiSeq 2000	RNA-seq	Gong et al., 2020
GSE28892	2011	*Mus musculus*	GPL10333 Agilent-026655	Microarray	Shin et al., 2011
GSE56734	2014	*Mus musculus*	GPL7202 Agilent-014868	Microarray	Ito et al., 2014
GSE25048	2011	*Homo sapiens*	GPL6947 Illumina HumanHT-12 V3.0	Bulk RNA-seq	Kim et al., 2011
GSE101133	2017	*Homo sapiens*	GPL20795 HiSeq X Ten	Bulk RNA-seq	Yan et al., 2017
GSE75141	2017	*Mus musculus*	GPL7202 Agilent-014868	Microarray	Wu et al., 2017
GSE105019	2019	*Homo sapiens*	GPL16791 Illumina HiSeq 2500	Bulk RNA-seq	Fu et al., 2019
GSE112330	2019	*Homo sapiens*	GPL16791 Illumina HiSeq 2500	Bulk RNA-seq	Xie et al., 2019
GSE124528	2019	*Homo sapiens*	GPL16791 Illumina HiSeq 2500	Bulk RNA-seq	Wang et al., 2019
GSE116113	2019	*Homo sapiens*	GPL20795 HiSeq X Ten	scRNA-seq	Fu et al., 2019

### The Cancer Genome Atlas (TCGA) and Human Protein Atlas (HPA)

To explore the stemness of key miRNAs, we also downloaded the normalized microRNA-seq data and corresponding clinical information of HCC patients from TCGA^3^. The differential expression analysis and survival analysis were performed in R. “*P* < 0.05” was considered statistically significant. Meanwhile, the expression pattern of 23 genes in a normal liver tissue was also explored in the HPA database^4^ and only those with a moderate or high expression were shown.

### Short Time-Series Expression Miner (STEM) Analysis

The bulk RNA-seq and microRNA-seq data of hBTSCs, hHpSCs, hHBs, and hAHeps were used to conduct STEM analysis using the STEM v1.3.13 (Ernst and Bar-Joseph, 2006). mRNAs and miRNAs were all stratified into different profiles based upon various expression patterns calculated by STEM analysis, respectively. The four stages of hepatic lineage, including hBTSCs, hHpSCs, hHBs, and hAHeps, were considered in different time points.

### Gene Ontology (GO) and Kyoto Encyclopedia of Genes and Genomes (KEGG) Analyses

To explore the biological function of the hepatic lineage-specific gene profiles, KEGG and GO enrichment analyses were performed using the clusterProfiler R package (Yu et al., 2012). To understand the potential function of miRNAs, the DIANA-miRPath v3.0 database, a miRNA pathway analysis web-server^5^, was also adopted in this work because it could quickly and efficiently predict the potential targets of miRNAs and run the KEGG pathway analysis (Vlachos et al., 2015).

### miRNA-Related Databases

In our present work, we utilized three databases, including the microRNA target prediction database (miRDB) (Chen and Wang, 2020), the experimentally validated microRNA-target interactions database (miRTarBase) (Chou et al., 2018), and TargetScan (Lewis et al., 2003), to predict the targets of 52 miRNAs and only those targets overlapped by them could be used for further study. Subsequently, the predictive results were reassured by two comprehensive and integrative function microRNA databases, involving miRWalk (Sticht et al., 2018) and DIANA-microT-CDS (Paraskevopoulou et al., 2013). *P* < 0.05 was considered statistically significant.

### Establishment of “TF-miRNA-Target” Regulatory Network

Experimentally supported interactions between lineage-related miRNAs and their regulating TFs were downloaded from the TransmiR v2.0 database, which contains 3,730 TF-miRNA regulations supported by experiments, covering ∼623 TFs, ∼785 miRNAs, and 1,349 publications (Tong et al., 2019). The construction of the “TF-miRNA-target” regulatory network for hepatic lineage was performed via the Cytoscape Java version 3.7.1^6^ software (Shannon et al., 2003).

### Principal Component Analysis (PCA) and 3 Dimension_PCA Analysis

3D_PCA analysis was performed on bulk RNA-seq, bulk microRNA-seq of hBTSCs, hHpSCs, hHBs, and hAHeps to examine the performance of the 23-gene signature. The scRNA-seq data of 251 hepatoblasts/hepatocytes was also exhibited by PCA analysis to show the time-course change of hepatic lineage.

### Gene Set Enrichment Analysis (GSEA) and Gene Set Variation Analysis (GSVA)

Gene set variation analysis (GSVA) was used to score the RNA-seq data of hBTSCs differentiation and bulk RNA-seq of Dlk + hepatoblasts/hepatocytes, and each sample/cell received a GSVA score (Hanzelmann et al., 2013). All the fetal liver development and cell reprogramming related datasets have been used to perform the GSVA score to validate the lineage-specific characteristics of the 23-gene signature. In addition, GSEA analysis was used to uncover the relationship between PTEN/PIK3R1 and stemness of liver-related single cells^7^. The number of random sample permutations was 1,000.

### Correlation Analysis

The correlation between miRNA and targets or among the 23-gene signature, stemness, and PI3K/AKT signaling pathway was calculated to identify the regulatory relation of 23 targets, 17 miRNAs, and one signaling pathway. In our present study, *P* < 0.05 was considered statistically significant. Due to the various dataset sources and sample size, the threshold of correlation coefficient was not defined, and the specific data were all shown inside the corresponding figures.

### Statistical Analysis

Statistically significant differences between samples are calculated by using the Student’s two-tailed *t*-test and results are presented in a heatmap, and asterisks were used to show the differential expression results. *P* values of less than 0.05, 0.01, 0.001, and 0.0001 were considered statistically significant and exhibited as “^∗^”, “^∗∗^”, “^∗∗∗^”, and “^****^”, respectively.

## Results

### 52 miRNAs Were Gradually Downregulated Following the Hepatic Maturation of hBTSCs via Its Involvement in the Stem Cells Pluripotency Signaling Pathway

**FIGURE 1:**
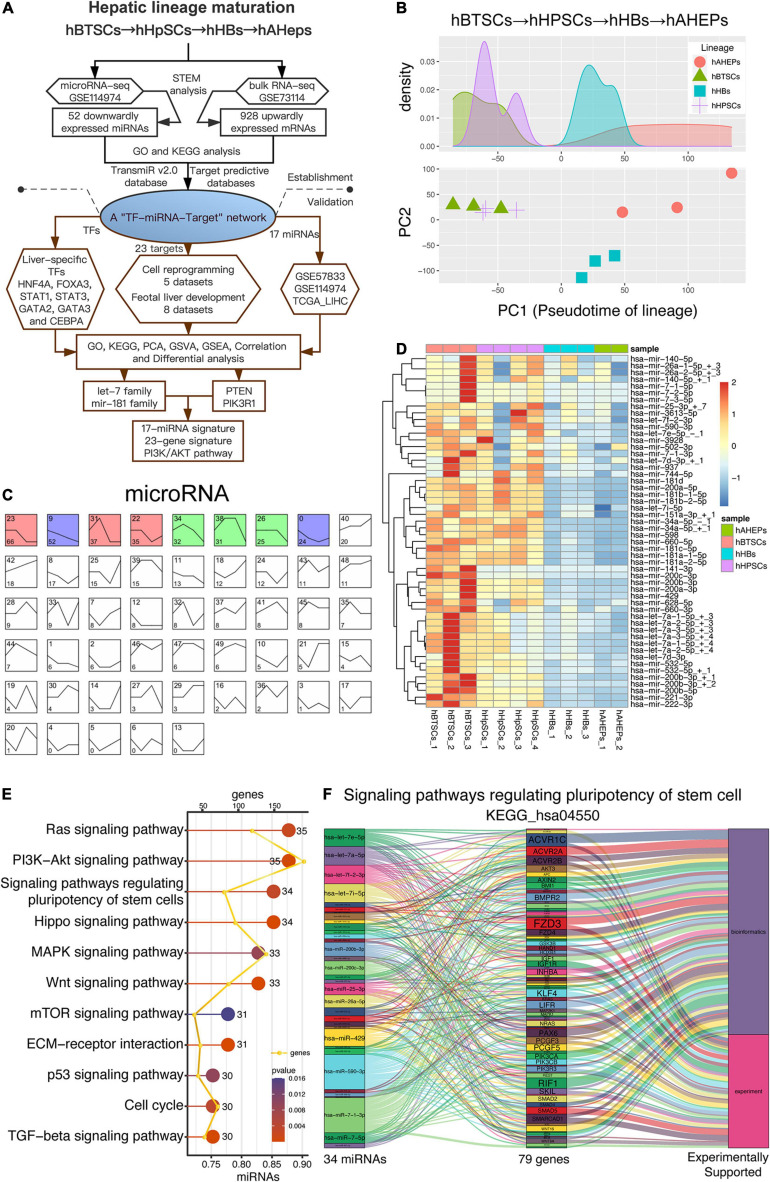
Downwardly expressed miRNAs during hBTSCs differentiation. **(A)** Flowchart of this study. **(B)** PCA analysis of four hepatic stages. **(C)** STEM analysis of the microRNA-seq data. Significant profiles are colored, and the same color represents a similar expression pattern. The top left number of each grid is the profile name while the bottom left number is the total significant genes included in this profile. **(D)** Heatmap of 52 miRNAs which are gradually downregulated from hBTSCs to hAHeps. **(E)** KEGG enrichment analysis of 52 miRNAs performed by the DIANA-miRPath v3.0 online database (http://snf-515788.vm.okeanos.grnet.gr). **(F)** The miRNA-target relationship in the signaling pathway KEGG_hsa04550, termed as the “Signaling pathways regulating pluripotency of stem cell.”

Our previous study has achieved the RNA-seq and microRNA-seq of four stages during the maturation of hepatic lineage, including hBTSCs, hHpSCs, hHBs, and hAHeps (Oikawa et al., 2015; Dinh et al., 2019, 2020). In order to explore the key microRNAs and mRNAs during the development from hBTSCs to hAHeps, bioinformatic analyses were performed in both the RNA-seq and microRNA-seq, and validation of the results have been comprehensively performed following our work flowchart (Figure 1A). As shown in Figure 1B, the PCA analysis has vividly plotted the developmental trajectory of these four stages. Based on these reliable data, STEM analysis was conducted. The microRNA-seq data were classified into 50 profiles and the significant ones, including the weight profiles, were colored (Figure 1C). Interestingly, we found that Profile 9 with a pattern that is straightly descending shows significance (*P* = 6.9E-16) from hBTSCs to hAHeps, while Profile 42 with the ascending trend was not statistically significant (Figures 1C,D), indicating that these 52 miRNAs in Profile 9 might exert critical functions during the maturation of the “hBTSCs-hHpSCs-hHBs-hAHeps” lineage.

To have a preliminary understanding of the function of these 52 descending miRNAs, the DIANA-miRPath v3.0 database, a miRNA pathway analysis web-server (see footnote five) was adopted (Vlachos et al., 2015). As shown in Figure 1E, the potentially involved signaling pathways of these 52 miRNAs were well-studied in stem cells, including the Hippo (Zhao et al., 2011), MAPK (Mossahebi-Mohammadi et al., 2020), Wnt (Sumi et al., 2008), and TGF-beta (Li and Wu, 2020) signaling pathways. Particularly, we noticed that the signaling pathway that regulates the pluripotency of stem cells (KEGG_hsa04550) was enriched (Figure 1E), with the involvement of 34 out of the 52 miRNAs (Figure 1F). Further evidences from bioinformatics or experiments with KEGG_hsa04550 indicated 79 genes participating in this pathway, including AKT3, IGF1, IGF1R, PIK3CA, PIK3CB, PIK3R3, WNT2, which are novel stem cells related genes that are important for the maintenance of stemness (Figure 1F and Supplementary Figure 1). As a result, the enrichment of the pluripotency of the stem cells (KEGG_hsa04550) signaling pathway enables us to confirm the indispensable role of these 52 miRNAs in hBTSCs (Figures 1E,F).

### A Total of 928 Genes Were Gradually Upregulated During the Maturation of the Hepatic Lineage

**FIGURE 2:**
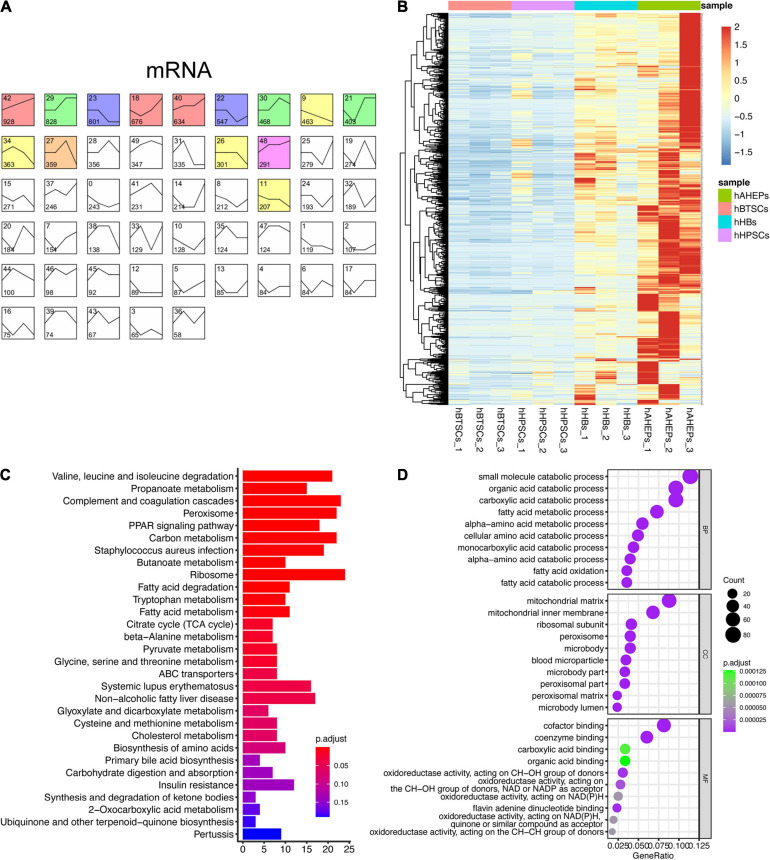
Upwardly expressed mRNAs during hBTSCs lineage commitment. **(A)** STEM analysis of the mRNA-seq data. Significant profiles are colored, and the same color represents a similar expression pattern. **(B)** Heatmap of 928 mRNAs which are gradually upregulated from hBTSCs to hAHeps. **(C)** KEGG enrichment analysis of 928 mRNAs using the package clusterProfier. Top 30 significant terms are shown. **(D)** GO enrichment analysis of 928 mRNAs using the package clusterProfier. Top 10 significant terms of biological process (BP), cell component (CC), and molecular function (MF) are shown, respectively.

Given that the expression pattern between mRNA and miRNA is usually negatively correlated (Chivukula and Mendell, 2008), we adopted the similar analytic strategy (STEM analysis) so that we could obtain the gradually ascending mRNAs, which might contain targets of the aforementioned 52 miRNAs. As shown in Figure 2A, the mRNA data were classified into 50 profiles and the only significant one, Profile 42, was colored. A total of 928 genes were obtained in Profile 42 with a trend of being gradually upregulated during the hepatic maturation (*P* = 6E-228, Figures 2A,B). KEGG and GO enrichment analyses were conducted to explore the functions of these 928 genes. Predictably, the results from the enrichment analyses all pointed to the metabolism-related pathways, which are critical for mature hepatocytes (Figures 2C,D).

### 23 Targets of miRNAs Were Focused Based on Several miRNA Target Predictive Databases

**FIGURE 3:**
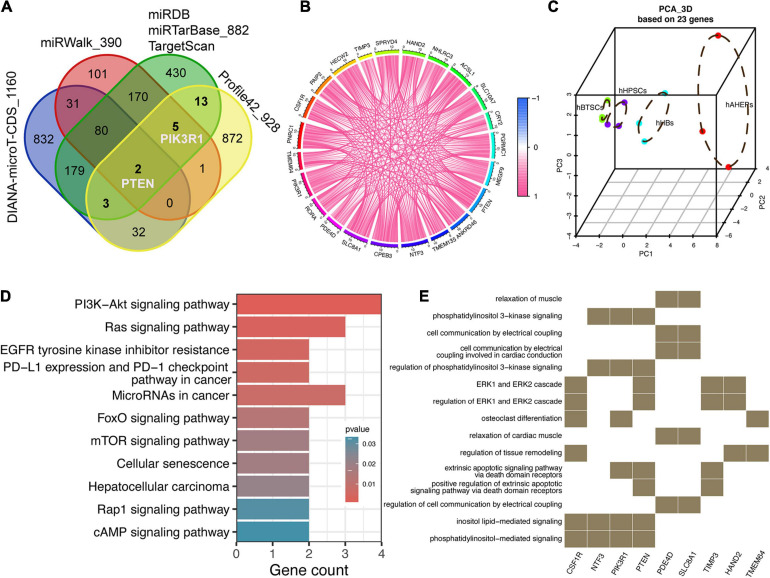
Prediction and function analysis of upwardly expressed targets according to the key miRNAs. **(A)** Venn plot of potential targets from five miRNA-related databases and 928 lineage-specific mRNAs. **(B)** Correlation analysis among 23 targets. **(C)** 3D PCA analysis of four stages of hepatic lineage based on 23 targets. **(D)** KEGG analysis of 23 targets. **(E)** GO analysis of 23 targets.

Provided that these 52 downwardly expressed miRNAs and 928 upwardly expressed mRNAs were lineage-specific during hBTSCs maturation into hAHeps, we then conducted more analyses to draw the possible interplay between them. Three traditional miRNA target prediction databases, including miRDB, miRTarBase, and TargetScan, were analyzed to explore the targets of these 52 miRNAs. Only those targets that were predicted by all of these three databases were selected for further analysis. With this stringent criteria, 882 targets were obtained (Figure 3A). After combining them with the 928 upwardly expressed mRNAs in Profile 42 from Figure 2A, 23 key targets, which were gradually upregulated with the “hBTSCs to hAHeps” development, were obtained. Then, we considered whether these 23 putative targets could be validated by two other comprehensive miRNA databases which also have a predictive function, miRWalk and DIANA-microT-CDS (Paraskevopoulou et al., 2013; Sticht et al., 2018). As a result, a total of 390 and 1,160 targets of our mentioned 52 miRNAs were predicted by the two databases, respectively (Figure 3A). As visualized in Figure 3A, two out of 23 targets (PTEN and ACSL1) were predicted by miRWalk and DIANA-microT-CDS. Another three out of 23 were found by the DIANA-microT-CDS database while five out of 23 were confirmed by the miRWalk database. In summary, PTEN and PIK3R1 were both included in these 10 genes, revealing them as reliable targets of miRNAs. PTEN and PIK3R1 were two critical genes involved in the PIK3/AKT signaling pathway, indicating a directly targeting signaling pathway regulation of those 52 miRNAs.

Intriguingly, we also found that the correlation among these 23 targets were rather close (Figure 3B) and the PCA analysis of only 23 targets could distinctly separate the samples into four cell types. Moreover, the distribution of the four cell types along the principal component 1 (PC1) matches the maturational trajectory from hBTSCs to mature hepatocytes (Figure 3C). From the human protein atlas (HPA), 13 out of 23 targets showed a moderate or high expression in the normal liver tissue (Supplementary Figure 2). On the other hand, the KEGG and GO analyses indicated that the PI3K/AKT signaling pathway was not only enriched by 52 miRNAs but also by these 23 targets (Figures 1E, 3D,E). Moreover, other signaling pathways that are closely related to the PI3K/AKT pathway, the Ras, mTOR, and EGFR tyrosine kinase inhibitor resistance pathways were all enriched (Yu and Cui, 2016). In conclusion, an indispensable role of the PI3K/AKT pathway during the development of hepatocytes was revealed according to our analysis (Figures 3D,E).

### A “TF-miRNA-Target” Regulatory Network of Hepatic Commitment Was Built Based Upon the TransmiR v2.0 Database and Cytoscape

**FIGURE 4:**
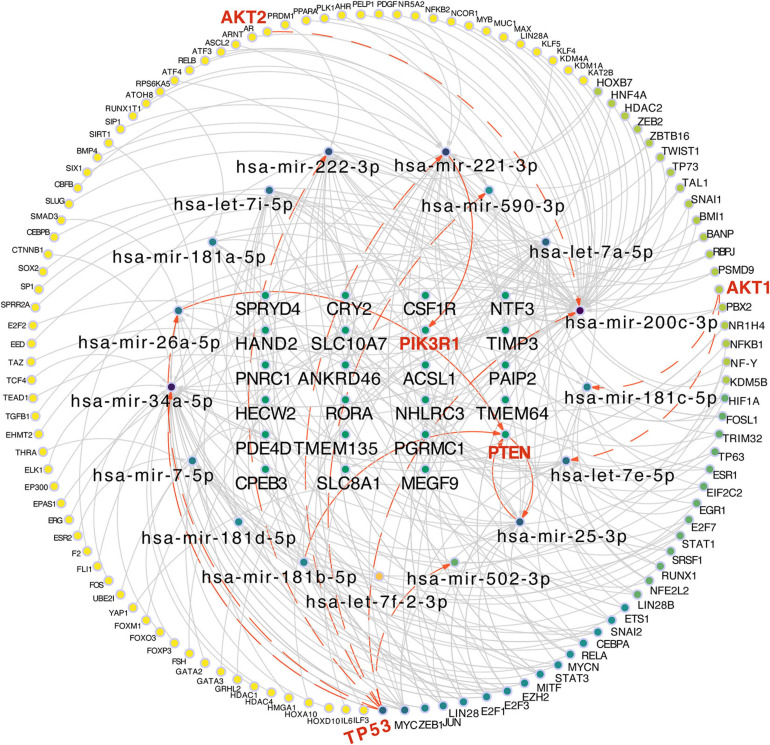
Construction of the “TF-miRNA-target” regulatory network based on 120 TFs, 17miRNAs, and 23 targets. Red and bold labels are the potential molecules included in the “PIK3/AKT” pathway. The line and arrow are the potential regulatory direction between linked nodes.

According to the target prediction databases, we extensively filtered the result of 52 miRNAs and 928 key genes, and finally narrowed it down to 17 key miRNAs and 23 targets which were closely related to the hepatic maturation. Considering that transcriptional factors are important in regulating the cell differentiation and lineage commitment, we intended to confirm the transcription factors that regulate the expression of miRNAs which are related to these 23 targets. Thus, the TransmiR v2.0 database was used to collect the TFs which were experimentally validated and considered capable of modulating these miRNAs. As a result, 120 TFs related to the 17 miRNAs were achieved and thereafter, the “TF-miRNA-target” regulatory network was constructed (Figure 4). Intriguingly, five of let-7 family and four of mir-181 family were included in these miRNAs (Figure 4), consistent with their potential regulation of stem cell differentiation and complicated relationship between let-7 and mir-181 (Koh et al., 2010; Li et al., 2012).

From other perspectives, various hepatic lineage-specific transcriptional factors, including HNF4A, FOXA3, STAT1, STAT3, GATA2, GATA3, and CEBPA, which have been applied in the studies of conversing other cell types into mature hepatocytes (Huang et al., 2011) or liver progenitor cells (Yu et al., 2013), were also able to modulate these 17 miRNAs (Figure 4). Therefore, the “TF-miRNA-target” regulatory network might indicate that these 17 miRNAs, especially the let-7 family and mir-181 family, were regulated by these lineage-specific TFs and affect the potential targets, thereby enhancing the maturation of the hepatic lineage. In addition, four TFs (AKT1, AKT2, TP53, PTEN) and two targets (PTEN and PIK3R1) were all included in the network, suggesting that these 17 miRNAs and 23 targets might affect the lineage commitment through the regulation of the PI3K/AKT pathway. Of note, PTEN not only served as a transcription factor for hsa-mir-25-3p, but also the target of hsa-mir-25-3p, hsa-mir-26a-5p, and hsa-mir-181b-5p, which implied a complicated feedback regulatory mechanism between mRNAs and miRNAs.

### Validation of 17 Lineage-Specific miRNAs Was Conducted During Hepatic Maturation

**FIGURE 5:**
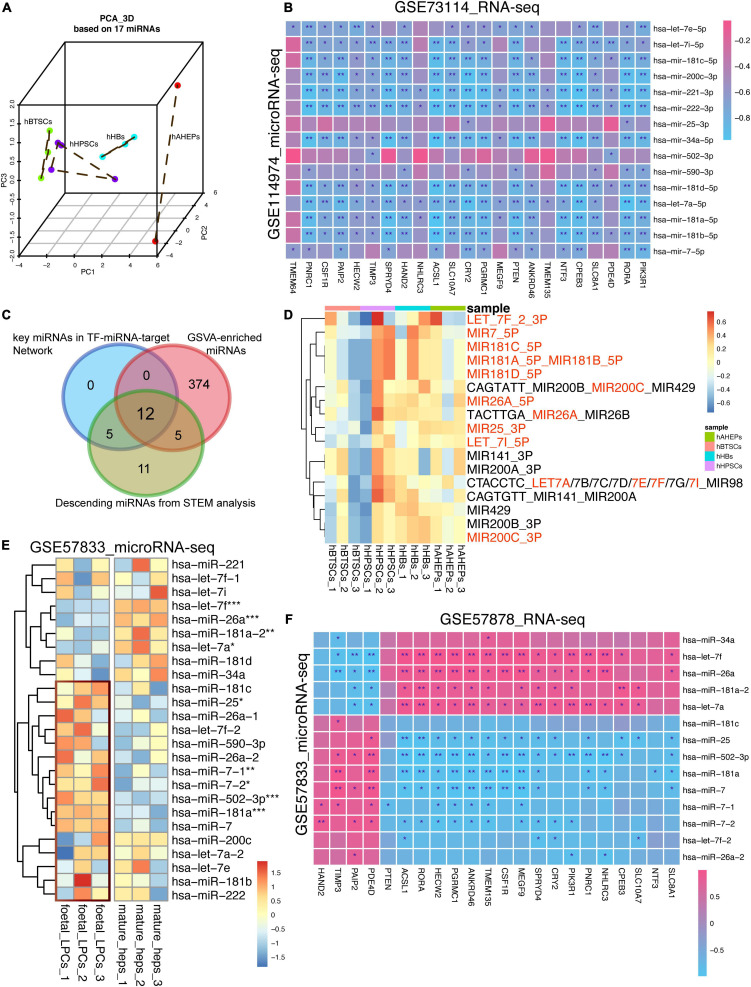
Validating the lineage-specific role of 17 key miRNAs in hepatic maturation. **(A)** 3D PCA analysis of four stages of hepatic lineage based on 17 miRNAs. **(B)** Correlation analysis based on the microRNA-seq (GSE114974) and RNA-seq data (GSE73114). *P < 0.05, **P < 0.01, and ***P < 0.001. **(C)** Venn plot of miRNAs obtained from different bioinformatic methods. **(D)** Expression profiling of 17 GSVA-enriched miRNA terms, which were also involved in the downwardly expression miRNAs obtained from STEM analysis. **(E)** Expression of 17 key miRNAs in GSE57833 dataset. *P < 0.05, **P < 0.01, and ***P < 0.001. **(F)** Correlation analysis based on the microRNA-seq (GSE57833) and RNA-seq data (GSE57878). *P < 0.05, **P < 0.01, and ***P < 0.001.

To confirm the lineage-specific characteristics of these miRNAs, PCA analysis based upon these 17 miRNAs was conducted. As shown in Figure 5A, the 12 samples of hBTSCs, hHpSCs, hHBs, and hAHeps could be separated albeit one sample of hHpSCs were slightly mixed with hHBs. Following PC1, all the samples were well ordered and consistent with the maturation stages developing from hBTSCs and hHpSCs to hHBs and hAHeps, indicating the time-course features of the miRNAs. In parallel, according to the correlation analysis based on the RNA-seq and microRNA-seq data of our samples, most miRNAs were significantly correlated with these 23 targets. Additionally, considering that most of the 928 upwardly expressed mRNAs might serve as targets of these 17 miRNAs, we also performed a GSVA analysis of these 928 mRNAs to verify whether these 17 miRNAs could be fetched according to the expression of these 928 mRNAs. As shown in Supplementary Figure 3A and Figure 5C, 408 miRNA terms archived in the molecular signatures database (MSigDB^8^) were enriched and most of them had an upwardly expressed pattern during hepatic development, revealing that the targets of the miRNA terms were gradually upregulated. Interestingly, 17 miRNAs were intersected with the downwardly expressed miRNAs and 12 of these 17 intersected miRNAs were involved in the regulatory network, which reassured the important role of these miRNAs via the GSVA analysis (Figure 5B).

The same GSVA analysis was conducted on another dataset (GSE90047) referred to as mouse embryonic hepatoblast to hepatocyte maturation, which had reported that 2,298 genes were gradually upregulated during the mouse fetal hepatoblasts differentiating to hepatocytes (Yang et al., 2017). Developed from the bulk RNA-seq data of these 2,298 genes, 1,403 miRNA terms from MSigDB were obtained by the GSVA analysis. Most of these GSVA-enriched miRNA terms were gradually upregulated from embryonic days 12.5 to 18.5 in spite of their high expression in embryonic days 10.5 to 11.5, indicating that the gene targets of miRNAs were gradually increased during the embryonic liver development (Supplementary Figure 3C). Intriguingly, all 408 GSVA-enriched terms found in our current study were included by the 1,403 terms of this dataset (GSE73114), affirming the potential lineage-dependence of our miRNAs (Supplementary Figure 3B). Herein, the same 17 GSVA-enriched miRNAs terms mentioned above (Figure 5D) were extracted for further study. These 17 miRNA terms were also gradually upregulated from embryonic days 13.5 to 18.5, though they were downregulated from embryonic days 10.5 to 13.5 (Supplementary Figure 3D).

Then, a microRNA-seq dataset (GSE57833) about fetal liver progenitor cells and mature hepatocytes was used to validate the results. Regardless of the distinct difference between our data and GSE57833 due to the batch effect, most of these 17 miRNAs were presented with an upregulated tendency in fetal liver progenitor cells rather than in mature hepatocytes (Figure 5E). With the combination analysis of its mRNA-seq dataset (GSE57878), most of the 17 miRNAs proved to be negatively correlated to the 23 targets (Figure 5F), which was in line with the correlation analysis results in Figure 5B. All in all, our study confirmed that these 17 key miRNAs were hepatic maturation-specific for hBTSCs.

### The Lineage-Specific Signature of 23 Key Genes Also Exists in Fetal Liver Development

**FIGURE 6:**
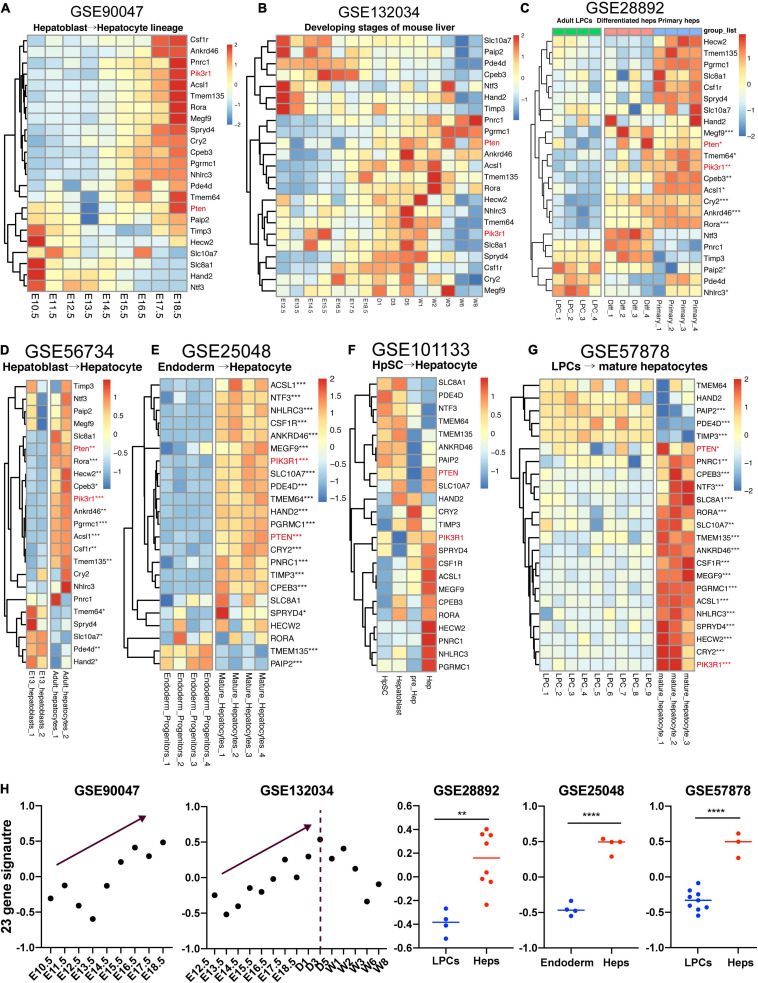
Expression pattern of 23 genes in fetal liver development. **(A–G)** LPC, liver progenitor cell; Diff, differentiated hepatocyte; Primary, primary hepatocyte; HpSC, hepatic stem cell; Hep, hepatocyte. **(H)** GSVA analysis of 23-gene signature across the datasets with enough samples. *P < 0.05, **P < 0.01, ***P < 0.001, and ****P < 0.0001.

Various researches have comprehensively depicted the developmental biology of fetal liver (Yang et al., 2017; Gong et al., 2020). However, whether these 23 genes obtained from our study could show the lineage-specific characteristics during fetal liver development remains ambiguous. Here, we gathered two datasets studying the mouse embryonic liver development and five datasets focusing on the differentiation of mouse or human liver-related stem cells, thereby enabling us to unveil the expression pattern of these 23 genes which were upwardly expressed from along the maturation of hBTSCs to hAHeps. Figure 6A shows the RNA-seq data (GSE90047) of Dlk^+^ hepatoblasts/hepatocytes sorting from E10.5 to E17.5 mouse embryos, representing the hepatoblast-to-hepatocyte differentiation in the fetal liver (Yang et al., 2017). Within the development of Dlk^+^ cells from E10.5 to E17.5, most of these 23 genes were gradually upregulated and consistent with the expression pattern in the hBTSC-to-hAHep differentiation. As for another dataset (GSE132034) of fetal liver development from embryonic day 12.5 to postnatal days 1, 3, 5, and to weeks 1, 2, 3, 6, and 8, a similar result as GSE90047 was obtained for stages of E12.5 to E18.5. However, with stages going on after postnatal 1 or 3 weeks, genes began to be gradually downregulated (Figures 6B,H), showing a similar pattern as the metabolism-related module_3 and module_4 which was exhibited in the work of Gong et al.’s (2020) team. In addition, other cell types in the whole liver organ might contribute to this different peak of score with the weight of liver growing fast after the postnatal weeks as Gong et al. (2020) has reported. In addition, a similar result was obtained by analyzing the expression profiling between E13 hepatoblasts and adult hepatocytes in another dataset (GSE56734) (Figure 6D). The dataset (GSE28892) about adult mouse liver progenitor cells (LPCs) and primary hepatocytes was used in further investigating the expression pattern of these 23 targets in mouse. As vividly shown in Figure 6C, most of these 23 genes were highly expressed in primary hepatocytes compared to LPCs and the *in vitro* differentiated hepatocytes from the LPCs also have a high tendency of expressing these 23 genes.

Now that the lineage-specific characteristics of the 23 vital genes also existed in mouse hepatic maturation, we further verified these 23 gene signatures in the other three human datasets. Firstly, our study has recently noticed that the early stage of hBTSCs were rather similar to the endoderm progenitor cells (data not shown). Thereafter, the dataset (GSE25048) associated with endoderm progenitor cells and mature hepatocytes was analyzed (Kim et al., 2011). Unsurprisingly, 21 out of 23 genes had a higher expression trend in mature hepatocytes than in the endoderm progenitor cells, and 18 of them showed significant statistics and most of the P-value were even smaller than 0.001 (Figure 6E).

Secondly, we also analyzed the RNA-seq dataset (GSE101133), which contains four samples as follows: hHpSCs, hHBs, hepatic precursor cells, and hAHeps. Albeit the tendency seemed ambiguous due to the lack of enough replicate samples, we could still identify that more than half of the genes were highly expressed in the mature cells rather than the immature cells, including PIK3R1, PTEN, RORA, ACSL1, CSF1R and so on (Figure 6F). Finally, another dataset containing fetal LPCs and mature hepatocytes was achieved. In parallel with the other two human datasets, 18 out of 23 genes were markedly upregulated in mature hepatocytes (Figure 6G). Taken together, the aforementioned results demonstrated that 23 genes were lineage-dependent during hepatic maturation (Figure 6H).

### 23 Genes Tended to Be Upregulated in the Mature Hepatocytes Compared to the Liver Progenitor-Like Cells

**FIGURE 7:**
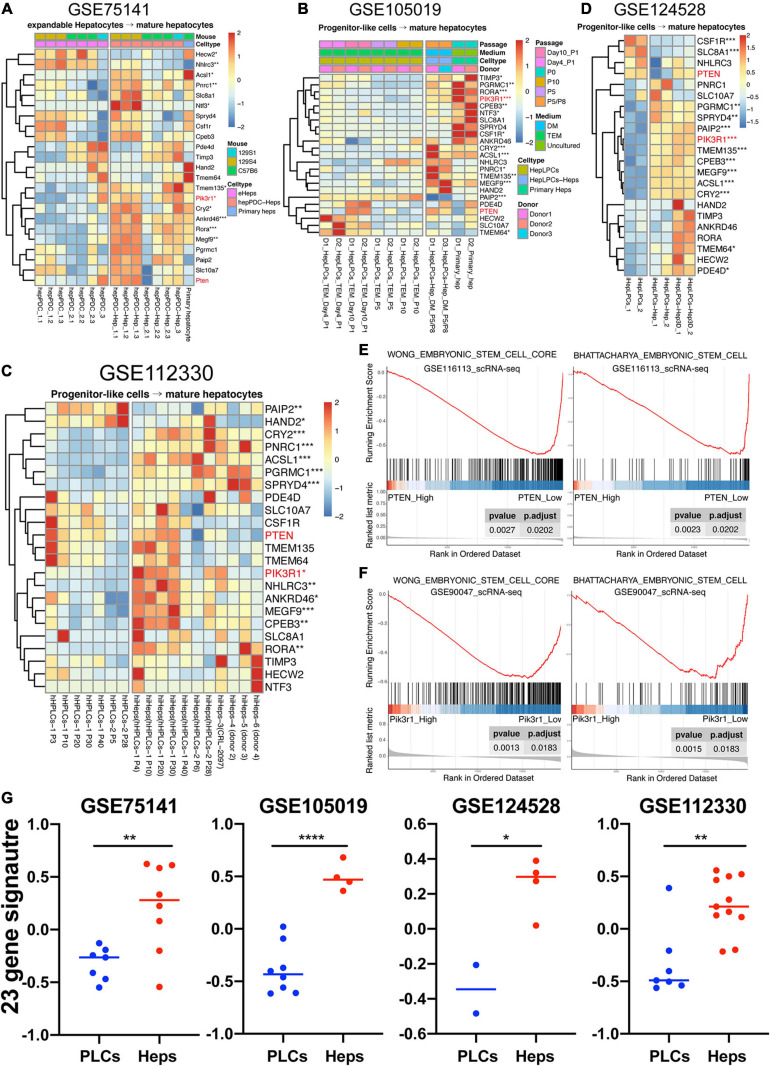
Highly expressed tendency of 23 genes in mature hepatocytes compared to liver progenitor-like cells. **(A–D)** hepPDC, hepatocyte-derived proliferative duct-like cell; hepPDC-Hep, hepPDC-derived hepatocyte; eHeps, expandable hepatocytes; heps, hepatocytes; HepLPCs, hepatocyte-derived liver progenitor-like cells; P, passage; hHPLCs, human progenitor-like cells; hiHeps, human induced hepatocytes; iHepLPCs, hepatocyte-induced liver progenitor cells. iHepLPCs-Hep3D, iHepLPCs organoid. **(E)** GSEA analysis of single cell datasets (GSE116113) based on PTEN expression. **(F)** GSEA analysis of single cell datasets (GSE90047) based on Pik3r1 expression. **(G)** GSVA analysis of 23-gene signature across the datasets with enough samples. *P < 0.05, **P < 0.01, ***P < 0.001, and ***P < 0.0001.

Studies conducted by us and other teams have demonstrated the possibility of reprogramming different cell types into progenitor-liked cells (Yu et al., 2013; Wu et al., 2017; Deng et al., 2018; Cheng et al., 2019; Fu et al., 2019; Wang et al., 2019). Here, two datasets of our previous work and two of others were collected to study the expression profiling of 23 genes in progenitor-like cells versus mature hepatocytes. One of our previous work focused on producing mouse expandable hepatocytes by reprogramming mature hepatocytes, which closely resembled duct-like cells and therefore named hepatocyte-derived proliferative duct-like cells (hepPDCs) (Wu et al., 2017). As shown in Figure 7A, the expression of the 23 genes had a lower tendency in hepPDCs rather than either the induced mature hepatocytes or primary hepatocytes. The lack of enough statistical significance and heterogeneous expression inner or between these different groups might be due to the various strains including 129S1, 129S4, and C57B6 mice. The other work was conducted to harvest the liver progenitor-like cells (HepLPCs) that were reprogrammed by human primary hepatocytes (Fu et al., 2019). Distinctly, despite the utility of different culture conditions, different donor samples, and even different passages, most of the 23 genes were significantly upregulated in the primary hepatocytes and induced mature hepatocytes (Figure 7B).

On the other hand, we also explored another research conducted by Xie et al. (2019), which uses a two-step lineage reprogramming strategy to generate functionally competent hepatocytes from fibroblasts. The RNA-seq data (GSE112330) of human hepatic progenitor-like cells (hHPLCs) and induced mature human hepatocytes (hiHeps) were obtained for further understanding the expression pattern of the 23 genes. Overall, the hiHeps were prone to express the 23 gene signature in contrast with hHPLCs, and 11 genes were significantly upregulated even with the various passages and donors (Figure 7C). Lastly, the dataset (GSE124528) containing the 3D culture of progenitor-like cells was also achieved (Wang et al., 2019). As displayed in Figure 7D, hepatic spheroids induced by the human hepatocyte-derived liver progenitor-like cells also highly expressed most of these 23 genes. As a result, our work demonstrated that the signature of these genes was lineage-specific (Figure 7G) and might be capable of serving as new markers for evaluating the efficiency and reliability of the reprogramming from somatic cells to LPCs.

### PTEN and PIK3R1 Were Novel Targets for the Lineage-Dependent miRNAs

According to the KEGG results based upon the 52 miRNAs or only well-predicted 23 targets (Figures 1E, 3C), the PTEN/PI3K/AKT signaling pathway was both enriched by them, indicating its potential involvement in the maturation of the hepatic lineage. Then, the PIK3R1 and PTEN were underlined in most of the prediction databases (Figure 3A), and PTEN could both act as the TF and target in the “TF-miRNA-target” regulatory network (Figure 4). Of note, the lineage-specific characteristics of PTEN and PIK3R1 were reassured not only in the fetal liver development but also in the reprogrammed LPCs lineage commitment (Figures 6, 7).

To validate the relationship between lineage differentiation and PTEN or PIK3R1, two scRNA-seq datasets (GSE116113 and GSE90047), including mouse and human species, were collected and the GSEA analysis was performed (Figures 7E,F). For one thing, our previous scRNA-seq data (GSE116113) of 7,459 progenitor-like cells (HepLPCs) were collected to infer the potential role of PTEN and PIK3R1 during hepatic differentiation (Fu et al., 2019). Intriguingly, the GSEA result of PTEN shows that the downregulation of PTEN led to promote the stemness of ESCs (Figure 7E). In addition, the GSEA result of PIK3R1 failed to show enough significance with these two terms (“WONG_embryonic_stem_cell_core” and “BHATTACHARYA_ embryonic_stem_cell”) (data not shown). For another, the scRNA-seq (GSE90047) of mouse fetal liver hepatoblasts/hepatocytes were used (Yang et al., 2017). Conversely, among these 251 fetal liver-related cells, the GSEA analysis result revealed that the high expression of Pik3r1 could inhibit cell stemness during mouse liver development (Figure 7F) while the GSEA analysis of Pten was not so significant (data not shown). Collectively, it is possible that either PTEN or PIK3R1 independently exerts their suppressive role along the maturational lineage even without collaboration.

### PI3K/AKT Pathway Was Gradually Suppressed With the Development of the Hepatic Maturational Trajectory

**FIGURE 8:**
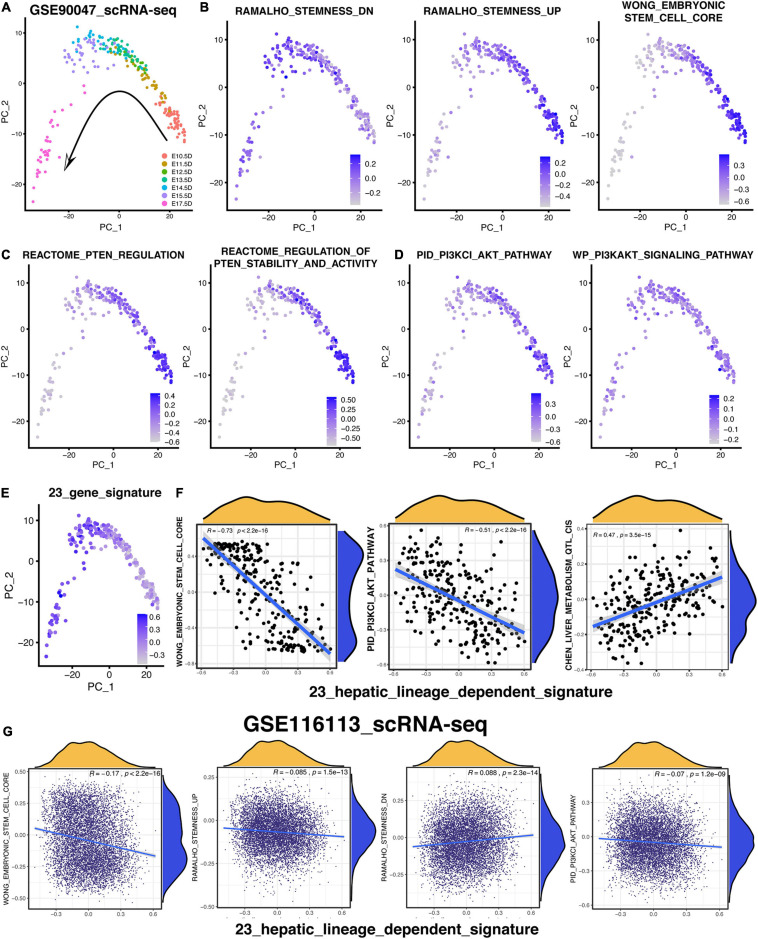
PTEN/PI3K/AKT signaling pathway is lineage-dependent activation following the hepatic maturational trajectory. **(A–E)** PCA analysis was used to exhibit the lineage trajectory of 251 hepatoblasts/hepatocytes. GSVA analysis was performed to understand various signature patterns of these scRNA-sea data. **(F)** Correlation analysis of 23-gene signature with others in 251 hepatoblasts/hepatocytes. **(G)** Correlation analysis of 23-gene signature with others in 7,459 hepLPCs.

To validate the relationship among the PI3K/AKT pathway, hepatic differentiation, and 23-gene-based signature, the scRNA-seq (GSE90047) of fetal liver hepatoblasts/hepatocytes were analyzed again (Yang et al., 2017). As vividly exhibited in Figure 8A, 251 hepatoblasts/hepatocytes were distributed exactly following the embryonic days based on the PCA analysis. Then, the GSVA analysis were performed to investigate various signatures of these single cells. According to the GSVA results in Figure 8B, the signature of “RAMALHO_stemness_down” was gradually increased while the signatures of “RAMALHO_stemness_up” and “WONG_embryonic stem cell” were gradually decreased. Therefore, the stemness of these hepatoblasts or hepatocytes was gradually downregulated following the time trajectory of the embryonic liver development and gradual development of hepatoblasts into hepatocytes (Figure 8B). Then, we focused on the time-course change of signatures related to the PTEN/PI3K/AKT pathway during hepatoblast-hepatocyte commitment. As shown in Figure 8C, the PTEN-regulated signatures were activated in earlier fetal cells, indicating that the activation of the PTEN/PI3K/AKT pathway was more likely to be in the hepatoblasts rather than the hepatocytes. Likewise, Figure 8D also reveals the same suggestion that the earlier the stages of the embryonic liver cells, the more active the PTEN/PI3K/AKT pathway is. In addition, five out of 23 genes, which were not included in the PTEN/PI3K/AKT pathway, also showed the distinctly upward expression tendency in the single cell level following the fetal hepatic development (Supplementary Figure 4C). Then, the hepatic-lineage-dependent signature constructed by the 23 genes was gradually upregulated during the trajectory (Figure 8E). Meanwhile, the 23-gene signature was positively correlated with the signature of liver metabolism and liver specific gene and negatively correlated with the stemness-related and PI3K/AKT-related signature, which was consistent with the results we figured out in the data of bulk RNA-seq (Figure 8F and Supplementary Figures 4B,D).

Considering that the correlation analysis that was only based on 251 mouse single cells obtained from Smart-seq2 technology might not be reliable enough, the 7,459 human HepLPCs, harvested by our team and sequenced by 10X Genomics technology, were collected to confirm the correlation result mentioned above (Fu et al., 2019). Similarly, the 23-gene signature was also positively correlated with the “RAMALHO_stemness_down” and “HSIAO_liver_specific_genes” and negatively correlated with the “RAMALHO_stemnness_up,” “WONG_embryonic stem cell,” “BHATTACHARYA_embryonic_stem_cell,” and “PID_PI3KCI_AKT_pathway” (Figure 8G and Supplementary Figure 4A). Most of the 7,459 hepLPCs had a similar pattern of these signatures with each other, indicating that most of the hepLPCs were in a stable and identical stage as what had been reported (Fu et al., 2019), thereby leading to the small correlation coefficients (Figure 8G and Supplementary Figure 4A).

In conclusion, the scRNA-seq result shows that the gradual inactivation of the PTEN/PI3K/AKT signaling followed the trajectory of the hepatoblast-to-hepatocyte commitment, which was consistent with the aforementioned hypothesis inferred by the enrichment analyses.

## Discussion

In recent years, many efforts have been made to improve the efficiency and elucidate the mechanism of hepatic commitment. However, most studies required an extensive manipulation of cells for obtaining seed cells. hBTSCs, as somatic stem cells located in the biliary tree, provide a different sight. The mRNA and protein level of how hBTSCs differentiates to hAHeps have been explored for a long time (Cardinale et al., 2011; Semeraro et al., 2012). However, it is unclear whether and how miRNAs, as epigenomic factors, regulate the hBTSCs-to-hAHeps transition. During hepatic regeneration, hBTSCs have been identified as the stem cells of hHpSCs or hHBs that gave rise to mature hepatocytes. In this study, we focused on the lineage-associated gene and miRNA expression during hepatic maturation utilizing the comprehensive bioinformatics analyses. Considering that the conversely dynamic changes in the miRNA and mRNA expressions during hepatic maturation might be vital, STEM analysis was applied to identify and further characterize significant time-series profiles. The identified time-series profiles and clusters will help in determining the miRNAs and targets regulating hepatic differentiation.

Notably, the downwardly expressed miRNAs and upwardly expressed mRNAs were detected, providing hints to the mechanistic understanding of hBTSCs-to-hAHeps transition. Given that Tsang et al. (2007) demonstrated that miRNAs could modulate the self-renewal and differentiation of ESCs through an integral biological network with TFs, we further combined our key miRNAs and mRNAs with experimentally validated TFs from the TransmiR v2.0 database. Thus, after filtering these key molecules through identifying their relationship using five miRNA-related databases and validating their lineage-dependent characteristics by more than 10 datasets containing microRNA-seq, bulk RNA-seq, and scRNA-seq, the “120_TF-17_miRNA-23_target” network for hepatic lineage was constructed. With the GO, KEGG, GSEA, and GSVA enrichment analyses, we speculated that this “120_TF-17_miRNA-23_target” biological network could affect the maturation stage of lineage cells by affecting the PI3K/AKT pathway.

We previously demonstrated the direct induction of functional hepatocyte-like cells from mouse tail-tip fibroblasts by the transduction of Gata4, Hnf1a, and Foxa3, and inactivation of p19Arf (Huang et al., 2011) while human induced hepatocytes (hiHeps) were generated from fibroblasts by FOXA3, HNF1A, and HNF4A (Huang et al., 2014; Yang et al., 2018). Then, Hnf1b and Foxa3 are sufficient to reprogram MEFs into the induced hepatic stem cells (Yu et al., 2013). Moreover, we also recently have reported that the combination of HNF1A, HNF4A, and FOXA3 synergistically reprograms the hepatocellular carcinoma (HCC) cells to hepatocyte-like cells (Cheng et al., 2019). Intriguingly, we could find that most of these TFs used in cell reprogramming is involved in the constructed network, thereby underscoring the reliability and applicability of our network and partly explains the mechanism of how the cells are reprogrammed using these TFs.

MicroRNA families refer to miRNAs of a given family sharing the sequence of the ‘seed region’ from the nucleotides two to eight at the 5′ end of the miRNA. miRNAs of the same family are considered to be capable of regulating the overlapping sets of target genes as the ‘seed region’ is particularly important for target specificity. Two miRNA families were highlighted from 17 lineage-related and downwardly expressed miRNAs in our studies, the let-7 family and mi-181family. It is note-worthy that five of the let-7 family were included in the network: let-7i-5p, let-7a-5p, let-7e-3p, let-7f-2-3p, and mir-7-5p (Figure 4). let-7 is able to target Dicer, which is the protein in charge for miRNA maturation (Mayr et al., 2007; Forman et al., 2008). Hence, we speculated that the let-7 family possibly served as a master regulator of itself or other miRNAs in the network and have a close relation with other miRNAs, especially the mir-181 family (Li et al., 2012). Additionally, Koh et al. (2010) has reported that the let-7 family of miRNAs was predominant in both the intra and extra cellular samples for MSC. HNF4A is indirectly regulated by the let-7 family in the undifferentiated mesenchymal stem cells (MSC) and HEPG2 cells (Koh et al., 2010). Interestingly, Peng et al. (2020) has showed that the upregulation of miR-25-3p in cardiomyocytes was sufficient to exert cardio-protective effects via a directly targeted PTEN both *in vitro* and *in vivo*. In addition, a feedback loop between miR-122 and most of the liver-enriched transcription factors, including HNF6, plays an important role in hepatic differentiation (Laudadio et al., 2012). Therefore, these might indicate the existence of a close and reciprocal loop between PTEN and miR-25-3p proposed in our network (Figure 4). Although further validation needs to be done both *in vitro* and *in vivo*, we could speculate that these 17 miRNAs, especially the let-7 family, might confer a certain function during hepatic maturation.

On the other hand, 23 genes were considered crucial due to their dual function: lineage-specific mRNAs with an upwardly expression and well-predicted targets of key miRNAs. Several enrichment analyses made us focus on PTEN and PIK3R1, two PI3K/AKT pathway suppressor genes. From our KEGG enrichment results in Figure 3C, it also showed that these 23 putative targets, which were predicted as miRNAs targets in our study, were involved in the “microRNAs in cancer” pathway, demonstrating the robust relationship between these 23 targets and their corresponding 17 miRNAs (Figure 3D). These also triggered us to consider whether these 17 miRNAs might function as the “oncogene” in malignancies. Intriguingly, we found that eight of these miRNAs were upregulated in the tumor tissue of hepatocellular carcinoma (HCC) and 12 of these miRNAs led to a worse prognosis for HCC patients (Supplementary Figures 5, 6). For example, mir-222, mir-221, mir-181a, mir-25, mir-7, and mir-502 were upregulated in a tumor tissue as well as predictive of a worse prognosis for HCC patients. Interestingly, several studies have revealed that the PTEN deletion would cause hepatopancreatic ductal malignancy and cholangiocarcinoma (Lin et al., 2018; Jiang et al., 2020). In addition, microRNA-181 has been identified as a vital player in EpCAM positive hepatic cancer stem cells (Ji et al., 2009). Our former studies have also reported that fibrolamellar carcinoma (FLC) cells were quite similar to hBTSCs (Oikawa et al., 2015). Therefore, the low expression of PTEN in hBTSCs might be reasonable and intelligible based on the similarity between the FLC and hBTSCs cells. These also indicated that the key miRNAs and targets in our work had a close relationship with the biliary tree cells and liver cancer cells.

While it is well established that the MAPK and HIPPO signaling are crucial for liver development (Zhao et al., 2011; Yang et al., 2017), little is known about how the PTEN/PI3K/AKT signaling could influence the LPCs differentiation. It has been reported that both PTEN and PIK3R1 (also known as PI3K p85α), a regulatory subunit of PI3K, are capable of inactivating the PI3K/AKT signaling and inhibiting tumor progression (Luo et al., 2005; Taniguchi et al., 2010; Vallejo-Diaz et al., 2019; Kong et al., 2020; Coleman et al., 2021). The tumor suppressor role of PIK3R1 was also validated in hepatocellular carcinoma (HCC) patients in TCGA analyzed by the Gene Expression Profiling Interactive Analysis (GEPIA) databases (Data not shown). Therefore, we supposed that the inactivation of PI3K/AKT signaling by PTEN and PIK3R1 was vital during the maturation of the hBTSCs-to-hAHeps lineage. Moreover, Wang et al. (2020) has recently reported that miR-100-3p targets PIK3R1 and suppresses the adipogenic differentiation of MSCs via the PI3K/AKT pathway. Li et al.’s (2019) team also suggests that human amniotic MSCs promote wound healing by enhancing cell proliferation through activating the PI3K/AKT signaling pathway (Li et al., 2019), which was consistent with our hypothesis in the context of hBTSCs-to-hAHeps transition. Thus, the PI3K/AKT pathway might also be important to maintain the stemness of hBTSCs and its inhibition could lead to hepatic maturation.

Future studies are needed to not only identify the miRNA and gene functions but also dissect the regulatory network for the pancreatic and cystic development of hBTSCs. There are some subjects to be overcome, which include the biological impacts of inhibitors of PTEN and PIK3R1 on the efficiency of hepatic differentiation of hBTSCs. Due to the complicated direct or indirect effect between the miRNA and targets, many experimental explorations are also needed to be conducted in the future. In addition, *in vitro* and *in vivo* works are needed to identify the prerequisite to improve the efficiency of generating BTSC-derived hepatic and pancreatic products for regenerative medicine. These data will be especially important for a succeeding *in vivo* post-grafting hepatic maturation of hBTSCs, when the functional mature hepatocytes are required by patients with liver injury within a relatively short time.

## Conclusion

We identified a group of potential miRNAs and putative targets associated with the hepatic differentiation of hBTSCs, thereby establishing the “TF-miRNA-target” regulatory network for better delineating the maturation of hepatic lineage. The well-defined biological features of hepatic-lineage cell types enabled the reliable bioinformatic analysis, while the published databases, from our or other studies referring to the hepatic maturation during fetal liver development and cell fate determination, provided sources for the validation. Diverse bioinformatic tools were applied to screen out a set of lineage-related miRNAs, especially the let-7 family, acting as suppressors during hepatic maturation mainly by regulating 23 targets and PI3K/AKT pathway. Further analysis revealed that among the 23-gene lineage-specific signature, PTEN and PIK3R1 might exert certain functions in regulating hepatic differentiation via the PI3K/AKT pathway. Our findings not only uncover the potential mechanism and functions of the regulatory network, but also discover possible strategies and factors which might predict or affect the functional maturation of hepatocytes both *in vitro* and *in vivo*.

## Data Availability Statement

The datasets presented in this study can be found in online repositories. The names of the repository/repositories and accession number(s) can be found in the article/Supplementary Material.

## Ethics Statement

This studies involving human participants was reviewed and approved by the Institutional Review Board for Human Research Studies at the UNC at Chapel Hill, NC, United States. The patients/participants provided their written informed consent to participate in this study.

## Author Contributions

XW conducted the overall analysis of the databases, designed the study, and prepared and revised every version of the manuscript with WZ, ZL, and ZH. HQ developed the bioinformatic analysis methods together with XW. TO, EW, and LR prepared the hBTSCs, hHpSCs, hHBs, and hAHeps cells and conducted the RNA-sequencing with PS. YY, JW, and LL ran the verification assays for forming the conclusions of this study. All authors contributed to the article and approved the submitted version.

## Conflict of Interest

The authors declare that the research was conducted in the absence of any commercial or financial relationships that could be construed as a potential conflict of interest.

## Footnotes

1 http://www.ncbi.nlm.nih.gov/geo

2 http://cran.r-project.org/web/packages/pheatmap/index.html

3 https://portal.gdc.cancer.gov

4 https://www.proteinatlas.org

5 http://snf-515788.vm.okeanos.grnet.gr

6 https://cytoscape.org

7 http://software.broadinstitute.org/gsea/

8 https://www.gsea-msigdb.org/

## Supplementary Material

Supplementary Figure 1 The specific regulated mechanism of KEGG_hsa_04550. The different color of each gene shows the potential number of their interacted miRNAs.

Supplementary Figure 2 Immunohistochemistry (IHC) of 13 genes in the HPA database.

Supplementary Figure 3 GSVA analysis of GSE73114 and GSE90047 bulk RNA-seq. **(A)** Heatmap of all GSVA-enriched miRNA terms according to GSE73114. **(B)** Venn plot showing the overlapped miRNA terms between GSE73114 and GSE90047. **(C)** Heatmap of all GSVA-enriched miRNA terms according to the bulk RNA-seq data from GSE90047. **(D)** The GSVA score level of 17 miRNAs along hepatic maturation.

Supplementary Figure 4 The characteristics of 23-gene signature in single cell levels. **(A)** Correlation analysis of 23-gene signature based on 251 cells. **(B)** Stemness features along hepatoblast-to-hepatocyte differentiation. **(C)** Expression pattern of five genes along the embryonic trajectory. **(D)** Correlation analysis of 23-gene signature based on 7,459 hepLPCs.

Supplementary Figure 5 Differential expression analysis of eight miRNAs based on HCC patients in TCGA.

Supplementary Figure 6 Survival analysis of 12 miRNAs based on HCC patients in TCGA.
