# Supplementary figures and images for: A MicroRNA-Based Network Provides Potential Predictive Signatures and Reveals the Crucial Role of PI3K/AKT Signaling for Hepatic Lineage Maturation
Source: Front Cell Dev Biol. 2021 Jun 1;9:670059. doi: 10.3389/fcell.2021.670059 (PMC8204022; doi:10.3389/fcell.2021.670059)

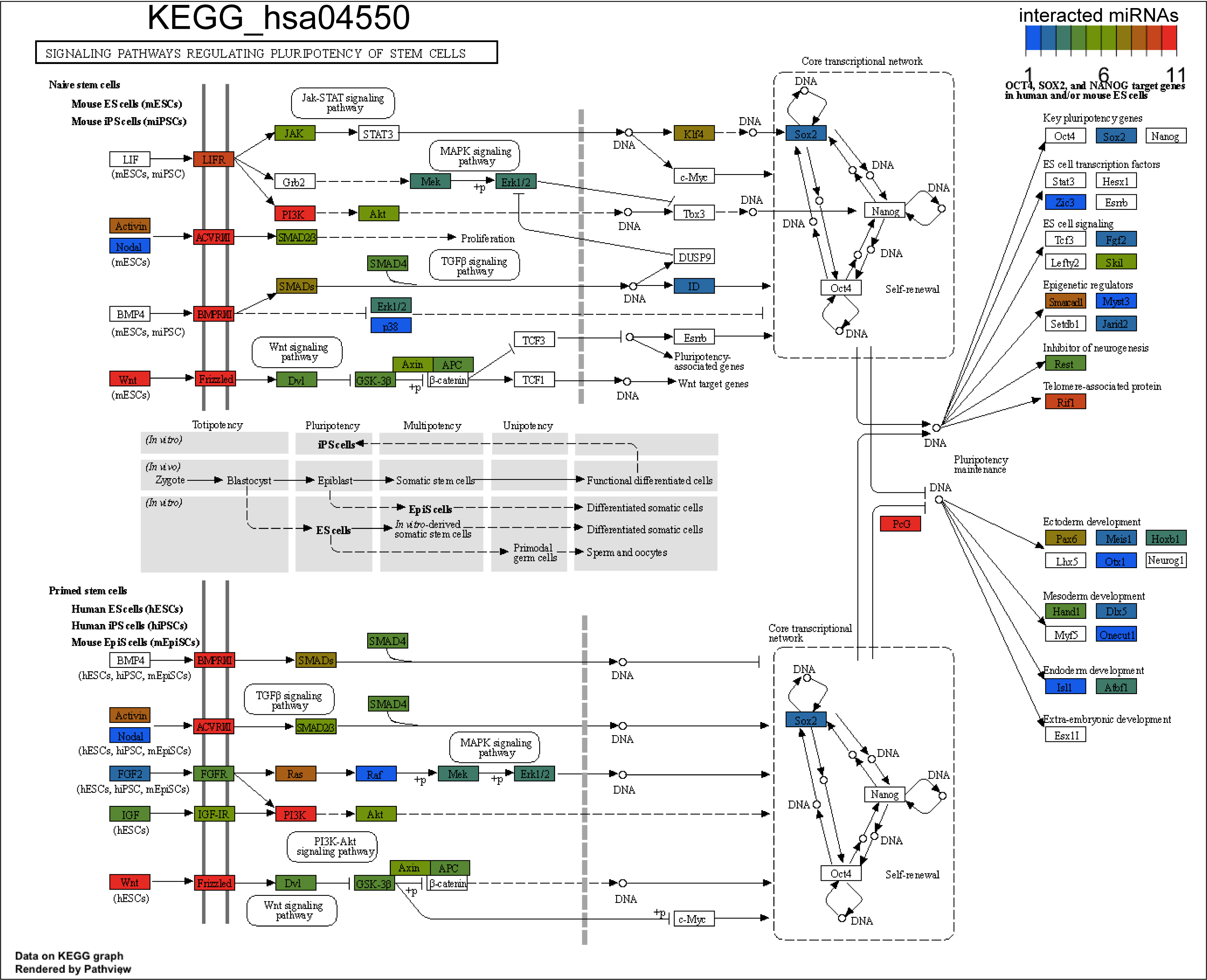

Supplement: Supplementary Figure 1 — The specific regulated mechanism of KEGG_hsa_04550. The different color of each gene shows the potential number of their interacted miRNAs. [file Image_1.TIF]

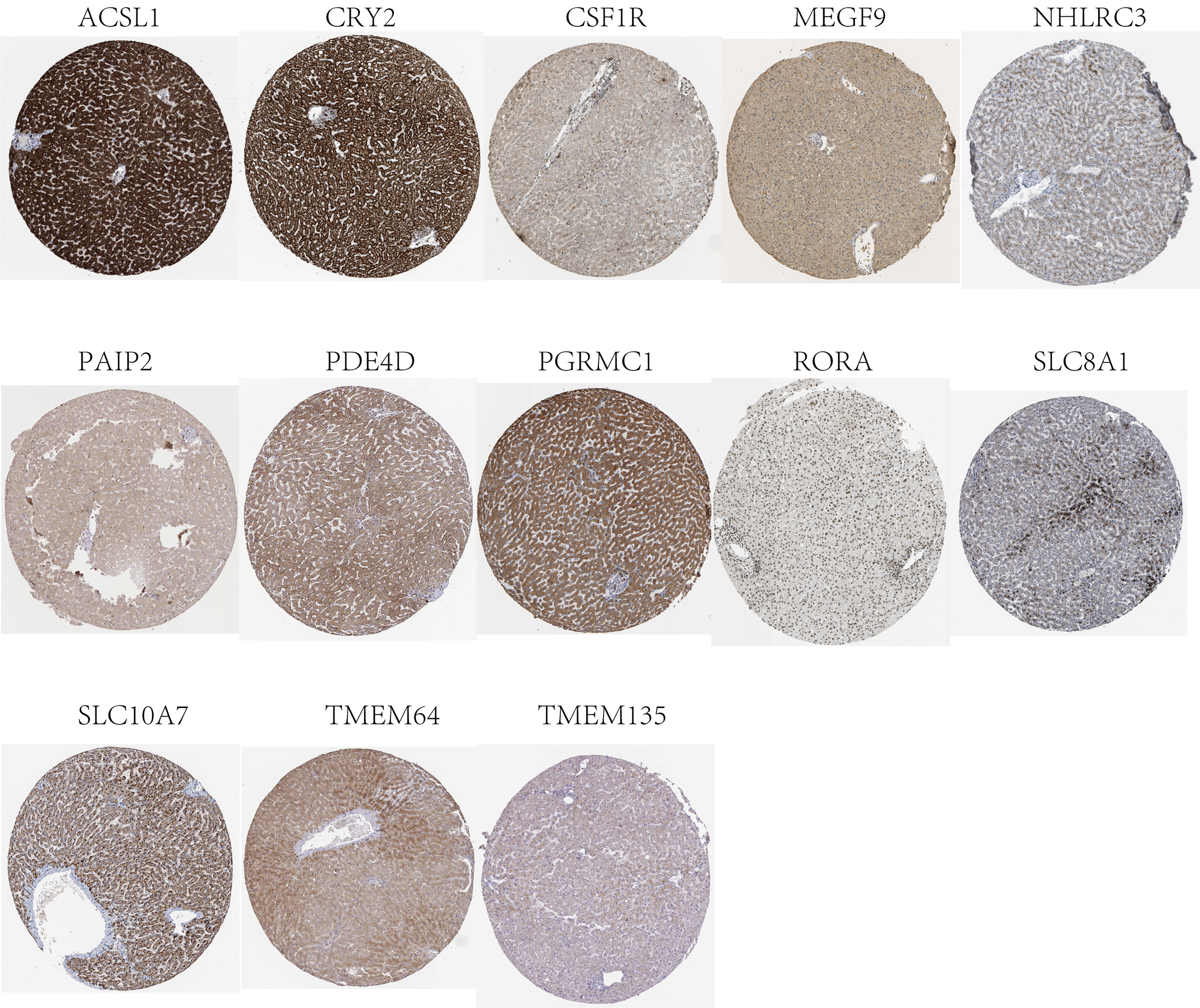

Supplement: Supplementary Figure 2 — Immunohistochemistry (IHC) of 13 genes in the HPA database. [file Image_2.TIF]

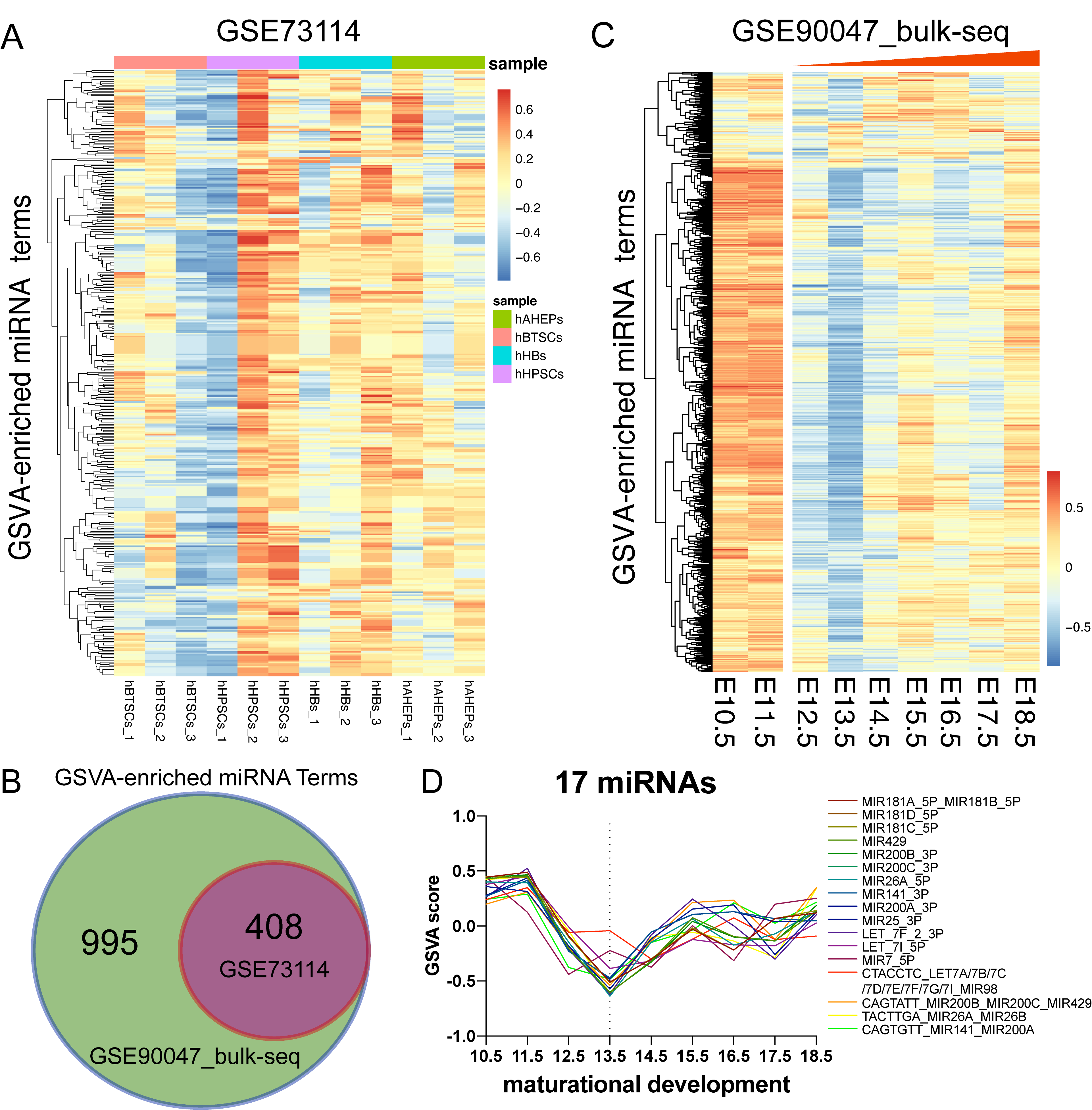

Supplement: Supplementary Figure 3 — GSVA analysis of GSE73114 and GSE90047 bulk RNA-seq. (A) Heatmap of all GSVA-enriched miRNA terms according to GSE73114. (B) Venn plot showing the overlapped miRNA terms between GSE73114 and GSE90047. (C) Heatmap of all GSVA-enriched miRNA terms according to the bulk RNA-seq data from GSE90047. (D) The GSVA score level of 17 miRNAs along hepatic maturation. [file Image_3.TIF]

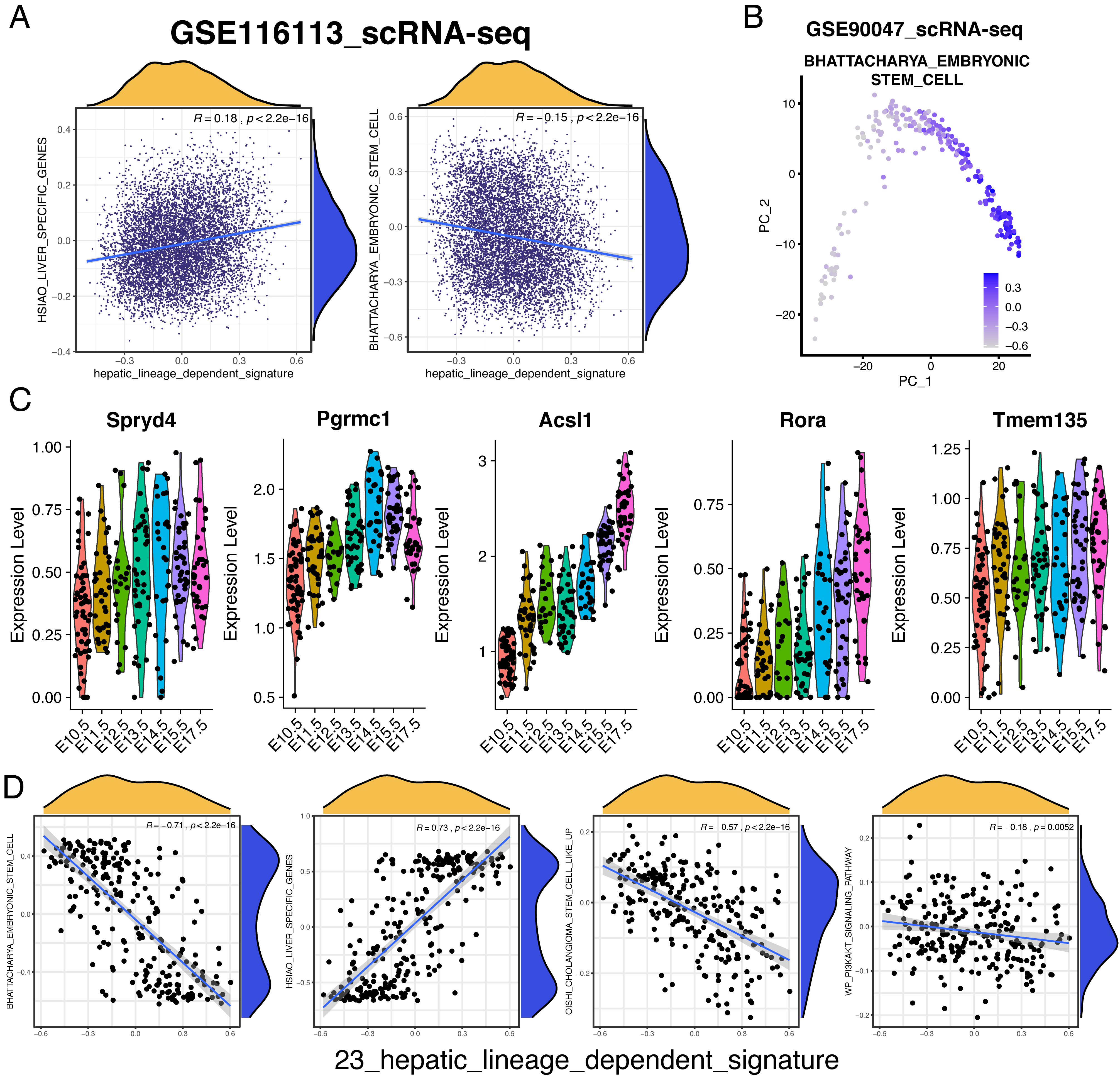

Supplement: Supplementary Figure 4 — The characteristics of 23-gene signature in single cell levels. (A) Correlation analysis of 23-gene signature based on 251 cells. (B) Stemness features along hepatoblast-to-hepatocyte differentiation. (C) Expression pattern of five genes along the embryonic trajectory. (D) Correlation analysis of 23-gene signature based on 7,459 hepLPCs. [file Image_4.TIF]

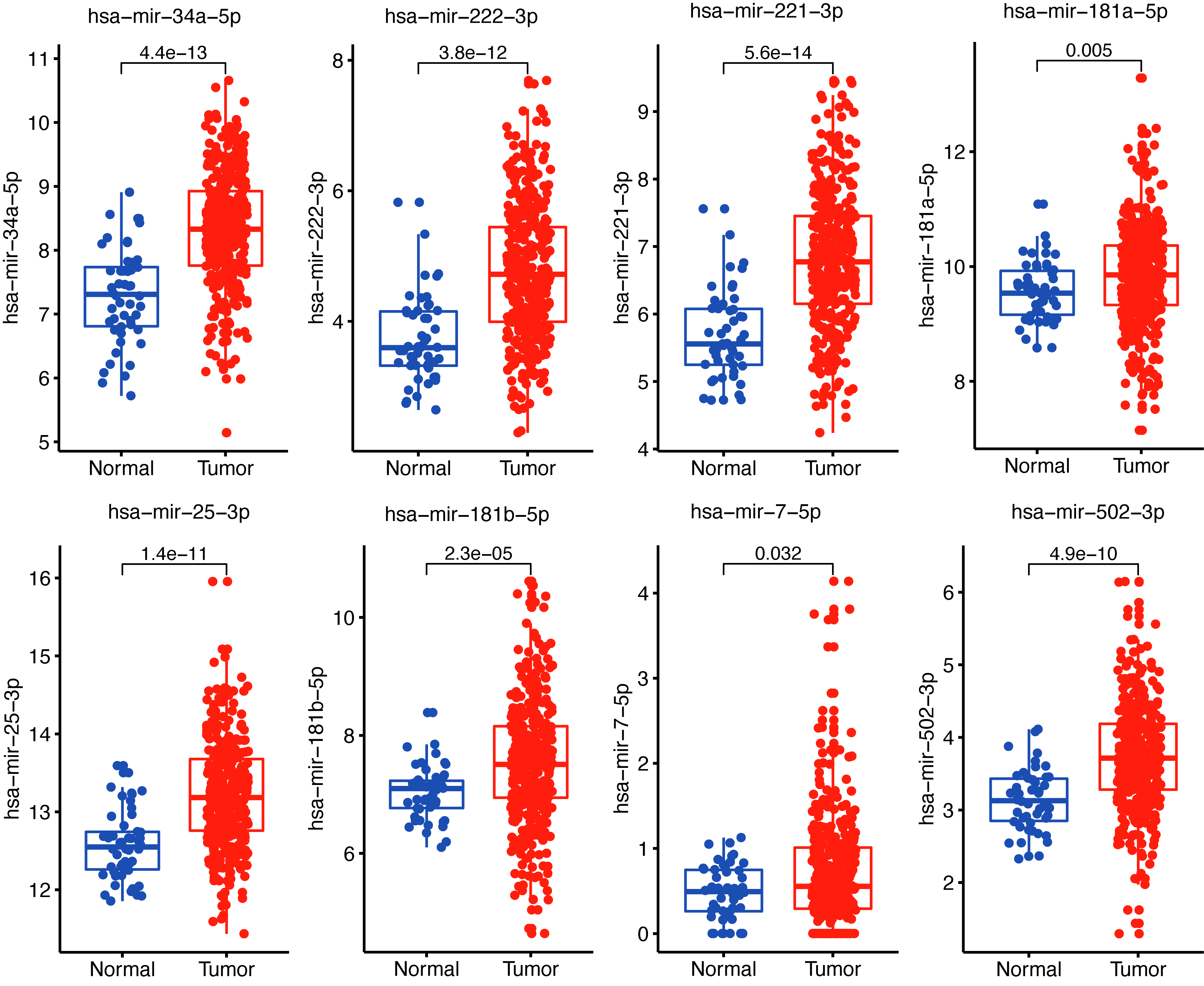

Supplement: Supplementary Figure 5 — Differential expression analysis of eight miRNAs based on HCC patients in TCGA. [file Image_5.TIF]

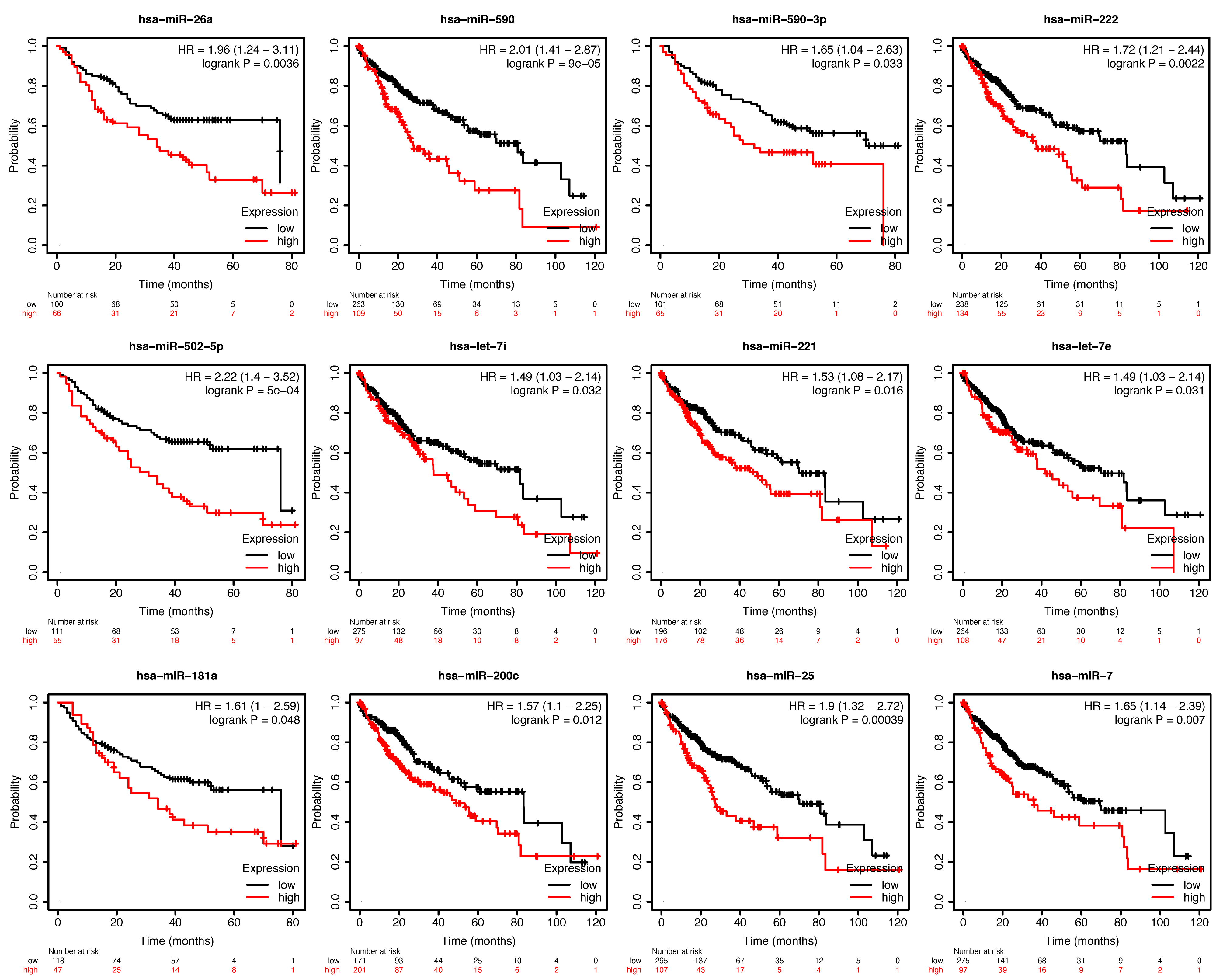

Supplement: Supplementary Figure 6 — Survival analysis of 12 miRNAs based on HCC patients in TCGA. [file Image_6.TIF]
